# Independent Band Modulation in 2D van der Waals Heterostructures via a Novel Device Architecture

**DOI:** 10.1002/advs.201800237

**Published:** 2018-08-02

**Authors:** Zhongxun Guo, Yan Chen, Heng Zhang, Jianlu Wang, Weida Hu, Shijin Ding, David Wei Zhang, Peng Zhou, Wenzhong Bao

**Affiliations:** ^1^ State Key Laboratory of ASIC and System School of Microelectronics Fudan University Shanghai 200433 China; ^2^ State Key Laboratory of Infrared Physics Shanghai Institute of Technical Physics Chinese Academy of science 500 Yutian Road Shanghai 200083 China

**Keywords:** 2D layered materials, MoS_2_, tunneling field‐effect transistors, van der Waals heterostructures, WSe_2_

## Abstract

Benefiting from the technique of vertically stacking 2D layered materials (2DLMs), an advanced novel device architecture based on a top‐gated MoS_2_/WSe_2_ van der Waals (vdWs) heterostructure is designed. By adopting a self‐aligned metal screening layer (Pd) to the WSe_2_ channel, a fixed p‐doped state of the WSe_2_ as well as an independent doping control of the MoS_2_ channel can be achieved, thus guaranteeing an effective energy‐band offset modulation and large through current. In such a device, under specific top‐gate voltages, a sharp PN junction forms at the edge of the Pd layer and can be effectively manipulated. By varying top‐gate voltages, the device can be operated under both quasi‐Esaki diode and unipolar‐Zener diode modes with tunable current modulations. A maximum gate‐coupling efficiency as high as ≈90% and a subthreshold swing smaller than 60 mV dec^−1^ can be achieved under the band‐to‐band tunneling regime. The superiority of the proposed device architecture is also confirmed by comparison with a traditional heterostructure device. This work demonstrates the feasibility of a new device structure based on vdWs heterostructures and its potential in future low‐power electronic and optoelectronic device applications.

## 1 Introduction

One‐ or few‐atom‐thick 2D layered materials (2DLMs), formed by lateral covalent bonds and vertical van der Waals (vdWs) forces, exhibit extraordinary electronic and optical properties^1^, ^2^, ^3^, ^4^, ^5^ and are therefore considered building blocks of next‐generation electronic devices. Recently, considerable research interest has been intrigued by the vertically stacked vdWs integration of various 2DLMs, which provides infinite possibilities by overcoming the limitation of lattice matching and processing compatibility.^6^, ^7^, ^8^, ^9^, ^10^, ^11^, ^12^, ^13^, ^14^ Among various categories of vertically stacked vdWs heterostructured devices, the tunneling field effect transistor (TFET), which provides a promising sub‐60‐mV dec^−1^ subthreshold swing (SS), has been regarded as a promising application of vdWs heterostructure for future energy‐efficient electronics.^15^, ^16^, ^17^, ^18^, ^19^

An effective gate control is crucial for the band alignment modulation in a TFET. Such a control capability is qualitatively correlated with the parameter called “natural length scale λ,” and hence requires an ultrathin thickness of channel materials.^20^ For bulk semiconductors (such as silicon, germanium, and III–V semiconductors), the quantum effects (such as bandgap variation and transconductance oscillation^21^) arise when thickness scales down to several nanometers, thus degrading the corresponding device performance.^22^ The band‐edge steepness at the heterojunction interface also plays an important role in obtaining devices with high switch steepness.^23^ Several factors affect the band‐edge steepness, including interfacial trap states, lattice mismatch, rough thickness, and doped atoms. In the case of ideal 2DLM heterostructures, the nature of atomic flatness and perfect surfaces enables the formation of atomically sharp interfaces without lattice mismatch, interface defects, and dangling bonds. Hence, the band‐edge steepness can, in principle, benefit from such high quality interfaces and it endows an abrupt tuning ability of density of states (DOS) at the band edge. Additional voltage potential dropped across the van de Waals gap^24^ also results in more effective modulation of band offset at the tunneling interface.

Consequently, significant efforts have been devoted to fabricating vertical stacked TFETs using different 2DLMs, such as graphene, hexagonal boron nitride (BN), and transition metal dichalcogenides (TMDs). Resonate tunneling current and negative differential resistance (NDR) have been observed in graphene‐based heterostructure TFETs, including monolayer and bilayer graphene separated by BN^25^, ^26^, ^27^, ^28^, ^29^, ^30^ or TMDs.^6^, ^31^ However, due to the absence of a bandgap, graphene‐based TFETs are unable to obtain a high on/off ratio of the current. TMD‐based TFET devices have also been experimentally demonstrated, including MoS_2_/WSe_2_,^24^, ^32^, ^33^ MoS_2_/BP,^34^, ^35^ SnSe_2_/BP,^36^ SnSe_2_/WSe_2_,^37^ and ReS_2_/BP.^35^ In these devices, gate‐tunable tunneling current governed by the band‐to‐band tunneling (BTBT) mechanism can be observed through electrostatic gating of energy‐band alignment and carrier density under specific device architectures. However, in the previously reported vertically stacked TFET devices, the gate electrical field is applied to both components of the heterostructure and thus modulates their band alignment simultaneously. Therefore, either an n‐ or p‐type 2DLM is under a depletion state when the device is turned on, which inevitably increases channel series resistance and limits the through current. In order to suppress the series resistance, recently, another self‐align method is proposed to fabricate short‐channel vdWs heterostructure device.^38^ It is also inefficient to modulate the band offset unless a more complicated architecture is used (e.g., dual‐gate structure^24^) or the gate‐control capability is drastically enhanced (e.g., by applying a thin, high‐k dielectric^24^ or ionic electrolyte^34^, ^39^). Moreover, in a vertically stacked 2DLM heterostructure, the energy‐band offset is mainly determined by the potential drop across the vdWs gap at the interface within the overlap region, and is difficult to be modulated directly by gate electrical field due to the screening effect associated with this vertically stacked structure.

Hence, in this paper, we design a novel top‐gated MoS_2_/WSe_2_ vdWs heterostructure by inserting a self‐aligned metal screening layer (Pd) on top of the WSe_2_ portion, which gives rise to a constant highly p‐doped state of the WSe_2_ while the independent energy‐band manipulation of the MoS_2_ can also be achieved. In such a device, a sharp PN junction forms at the border of the overlapped region and can be modulated efficiently by the gate electrical field. Under specific drain bias voltage *V*_D_, the device can be operated as a rectifier or BTBT diode, and gate modulation of drain current can be achieved in both operation modes. In the BTBT mode, high on/off ratio (>10^6^), high gate‐coupling efficiency (as high as 90%), and a small subthreshold wing (≈52 mV dec^− 1^) are obtained, indicative of efficient gate control on the MoS_2_ channel and effective electrical‐field screening on the WSe_2_ portion. We also compare the proposed new device architecture with a traditional heterostructure device to verify the superiority of its architecture. At low temperature, we also observe a novel gate‐dependent current fluctuation, which can be explained by the alternation of two different BTBT processes.

## 2 Device Structure and Fabrication

**Figure 1:**
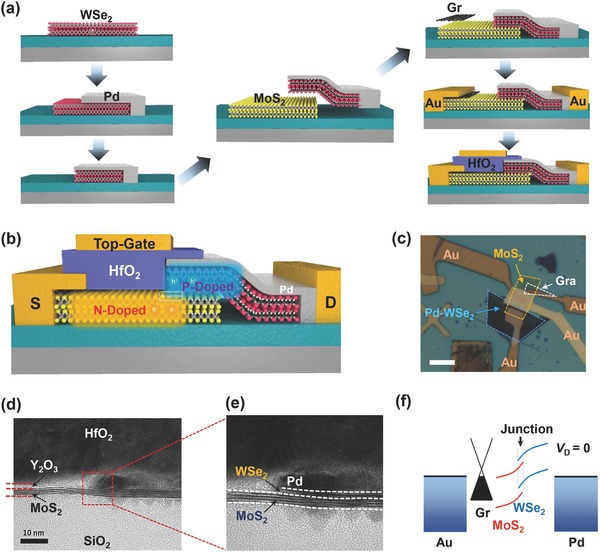
a) Schematic of fabrication process of the proposed MoS_2_/WSe_2_ heterostructure device. b) 3D schematic view of the heterostructure device and the illustration of the P‐ and N‐doped regions. c) Optical image of one MoS_2_/Pd‐WSe_2_ structured device on 300‐nm SiO_2_ substrate with 50‐nm top HfO_2_ dielectrics. Scale bar is 10 µm. d,e) Cross‐sectional HRTEM image of the heterostructure device and zoom‐in of the self‐aligned Pd/WSe_2_ portion. f) Schematic lateral band diagram of the MoS_2_/WSe_2_ device when *V*_D_ = 0 V.

The fabrication process and cross‐sectional structure of the proposed MoS_2_/WSe_2_ device are schematically shown in **Figure** **1**a (also see Experimental Section). Compared to a traditional heterostructure device, the main difference is the self‐aligned metal film (Pd in the proposed device) inserted between the HfO_2_ dielectric and the WSe_2_ sheet, which acts as contact and screening layer to the WSe_2_ portion, as shown by the high‐resolution transmission electron microscopy (HRTEM) images in Figure 1b,c, where a sharp edge at the Pd/WSe_2_ end can be clearly identified. Here, several reasons are considered for employing such screening metal film: 1) the Pd metal with large work function can completely deplete the electrons in the ultrathin WSe_2_, which consequently pulls down the Fermi level and further keeps the WSe_2_ a consistent p‐doped state. By gating the channel material (MoS_2_) only, it ensures an efficient modulation of energy‐band offset for such heterostructure. 2) Such structure enables the scale‐down of the TFET channel length, because one of the components (e.g., WSe_2_ in the proposed device) is totally covered under the screening metal film, which suggests that the device channel length is the same as in regular 2DLM‐FETs. Furthermore, to eliminate the Fermi pinning effect at the MoS_2_–metal interface, the contact of the proposed device can be improved by transferring graphene onto the MoS_2_ end, which improves contact resistance and enables more flexible modulation of the MoS_2_ energy band by the gate electrical field.

## 3 Results and Discussion

**Figure 2:**
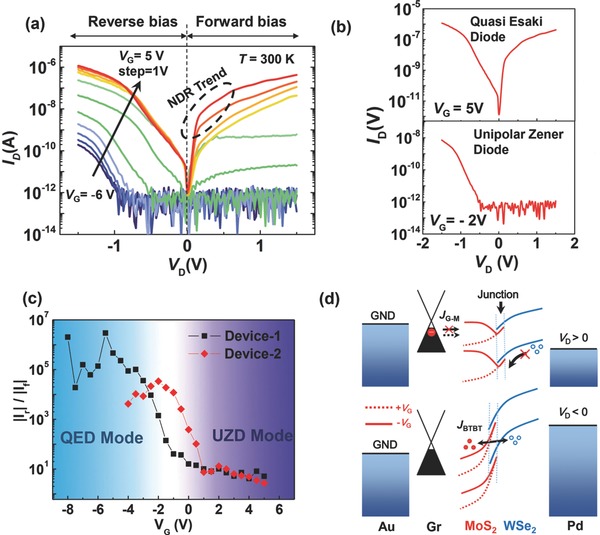
a) Output characteristics measured with *V*_D_ ranging from −1.5 to 1.5 V, and *V*_G_ varied with 1 V increments from −6 to 5 V. b) Output characteristics of QED regime (top) and UZD regime (bottom). c) Extracted |*I*_r_|/|*I*_f_| as a function of applied *V*_G_ from two devices. d) Energy‐band diagram of the proposed device with different work principles. Modulated band alignment under negative and positive *V*_G_ is plotted by solid and dashed lines, respectively. *J*_G‐M_ indicates the current from graphene to MoS_2_ and it is suppressed under negative *V*_G_ due to raised MoS_2_ band.

First, the MoS_2_/WSe_2_ heterojunction devices were electrically characterized using a probe station (Cascade SUMMIT 11000B‐M) at room temperature. **Figure** **2**a shows typical gate‐dependent output characteristics (*I*_D_–*V*_D_) with gate voltage *V*_G_ varied from −6 to 5 V in steps of 1 V. The different diode regimes can be observed by tuning *V*_G_. First, an obvious bipolar characteristic can be observed at *V*_G_ = 5 V (the upper graph of Figure 2b), which can be classified as a quasi‐Esaki‐diode (QED). It is indicative of the formation of a well‐stacked, highly doped p–n junction with a broken‐gap band offset (type‐III band alignment). In the proposed device architecture, the additional Pd film not only acts as a screening layer, but also as the contact metal that induces p‐doping of the WSe_2_ portion. Meanwhile, the positive *V*_G_ can effectively tune the MoS_2_ into an n‐doped state. Compared to a traditional Esaki diode, no obvious NDR effect is observed under forward bias, but with a trend as shown in the circled region in Figure 2a. We attribute this discrepancy to the traps in the WSe_2_ (tungsten or selenium vacancies) induced mainly by the etching process during device fabrication, which has also been previously reported in the WSe_2_–graphene heterostructures.^40^ These acceptor‐like traps^41^ provide an additional current component that is proportional to forward bias, through trap‐assisted tunneling. Second, when a negative *V*_G_ is applied (*V*_G_ = −2 V in the lower graph of Figure 2b), the minority carrier diffusion current under the forward bias *V*_D_ can be effectively suppressed, while a reverse tunneling current remains, showing a strongly rectified characteristic. Although the underlying transport mechanism is different, the measured *I*–*V* characteristics resemble the behavior of a unipolar Zener diode (UZD), which can be hardly observed in a single‐gated PN‐junction structure.

The reverse‐to‐forward drain current ratio (|*I*_r_|/|*I*_f_|, i.e., the rectified ratio) is then extracted from the fixed *V*_D_ = ±1.5 V, as shown in Figure 2c (result from a separate device is also added). The two operation regimes can be clearly identified, and the transition occurs at approximately *V*_G_ = −2 V, which also agrees with the n‐doped nature of MoS_2_. Such behavior can be quantitatively illustrated by the energy‐band diagram shown in Figure 2d. As the WSe_2_ is totally covered by Pd metal, only the graphene–MoS_2_ portion can be effectively modulated via the gate electrical field. For the QED mode (*V*_G_ > 0), four components contribute to the current at a forward *V*_D_: BTBT current (*J*_BTBT_), trap‐assisted tunneling current (*J*_TAT_), thermal emission current (*J*_Ther_), and interlayer recombination current (*J*_Re_). Although the occurring of *J*_Re_ is common in vdWs heterostructures due to strong Coulomb interaction,^24^, ^42^, ^43^ the magnitude of *J*_Re_ is negligible compared with the total current *I*_D_ under small positive *V*_D_.^43^ With a negative *V*_D_, in contrast, the current is mainly contributed by *J*_BTBT_. Under the UZD mode (*V*_G_ < 0) with a positive *V*_D_, electron diffusion current from graphene to MoS_2_ (*J*_G‐M_) is suppressed by a large Schottky‐barrier at the MoS_2_–graphene interface as MoS_2_ has been depleted by negative *V*_G_, similar to the OFF state in a graphene–MoS_2_–graphene transistor.^24^ The depleted state of MoS_2_ also suppresses *J*_Re_ as *J*_Re_∝*n*·*p*. The energy barrier at the MoS_2_–WSe_2_ interface blocks the hole diffusion path from the WSe_2_ portion due to the large bandgap of MoS_2_. Such transport mechanism can also be confirmed from n‐doping nature of MoS_2_–graphene barristor^43^ where the p‐branch is not observed. For a negative *V*_D_, the lifted MoS_2_ band requires a larger reserve bias to turn on the BTBT current.

**Figure 3:**
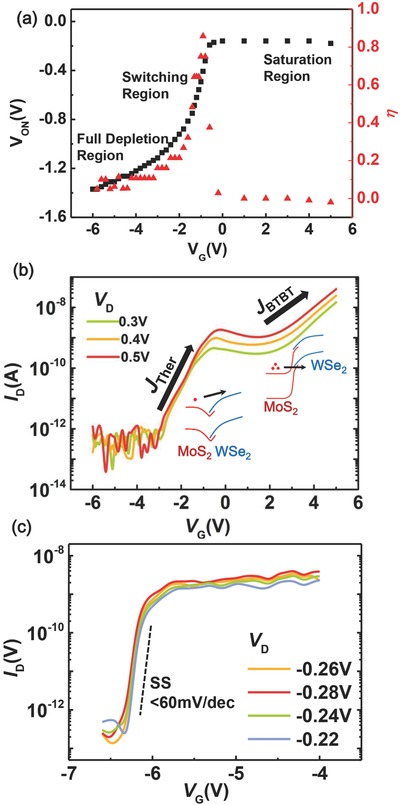
a) Extracted tunneling onset voltage *V*_ON_ (left Y‐axis, black squares) as a function of *V*_G_, with the slope representing the gate‐coupling efficiency η (right Y‐axis, red triangles). *V*_ON_ is defined by the reverse bias *V*_D_ when *I*_D_ reaches 100 pA. b) Transfer characteristics under positive *V*_D_ show obvious current fluctuations which are illustrated by corresponding schematic band diagrams. c) Transfer characteristics under negative *V*_D_ showing a sub‐60‐mV dec^−1^ SS over two decades.

We next examine the gate‐coupling efficiency of the proposed device to evaluate its gate‐control capability on relative band alignment between MoS_2_ and WSe_2_, which can be conveniently elucidated by the tunneling onset voltage *V*_ON_ (extracted from the *I*_D_–*V*_D_ curves and defined as the tunneling onset voltage *V*_ON_ at which the corresponding drain current *I*_D_ approaches 100 pA). The extracted *V*_ON_ versus *V*_G_ curves are shown in **Figure** **3**a. The nonlinear *V*_ON_–*V*_G_ curve indicates that the gate‐control capability can be enhanced by this novel device architecture, and only depends on the MoS_2_ channel. The slope of *V*_ON_–*V*_G_ represents the efficiency of the gate‐induced band shift between MoS_2_ and WSe_2_, and is thus defined as gate‐coupling efficiency η,^24^ which is noted by red points in Figure 3a. It is as high as 90% and comparable to that of dual‐gate architecture.^24^ We expect that the η could be further improved by employing thinner high‐k dielectrics. At larger *V*_G_, the electron screening effect, a result of the large DOS at the MoS_2_ conduction‐band edge, is the main reason for the saturation of *V*_ON_. This is also a proof of achieving independent manipulation of the MoS_2_ energy band, since such a saturation tendency has not yet been reported in traditional vdWs heterostructure devices, because in regular devices, the gate‐induced electrical field acts on both of the stacked materials, as discussed above.

Figure 3b shows the transfer characteristics (*I*_D_–*V*_G_) under positive *V*_D_. When applying a large negative *V*_G_, the MoS_2_ portion is fully depleted while WSe_2_ maintains p‐doped, which can be regarded as an I–P junction. In this regime, *I*_D_ is below the noise level of our measurement setup. Upon increasing *V*_G_, electrons tend to accumulate in the MoS_2_ channel, resulting in an enhancement of *I*_D_ (denoted as *J*_Ther_ in Figure 3b) which is the same as the subthreshold regime of a regular MoS_2_ FET. When the *V*_G_ continues to increase, an obvious current fluctuation is observed. This is mainly due to a higher energy barrier results from enhanced built‐in electrical field between MoS_2_ and WSe_2_, thus diminishing *I*_D_. By further applying a larger *V*_G_, the Fermi level shifts closer to the conduction‐band edge of MoS_2_, resembling an n^+^/p^+^ diode, and the BTBT window is now opened for electrons to tunnel from the MoS_2_ conduction band to the WSe_2_ valence band.

In order to demonstrate the feasibility of the proposed device working as a TFET, we also measure its transfer characteristics under negative *V*_D_. The best performance from a separate device is plotted in Figure 3c with different *V*_D_ values. A sharp switching of *I*_D_ is observed, and the on/off ratio of the device exceeds 10^4^. The SS of the heterojunction device is calculated according to SS = d*V_G_*/d (log *I_D_*). A low SS of 52 mV dec^−1^ was obtained and maintained sub 60 mV dec^−1^ for two decades of *I*_D_ from 10^−10^ to 10^−8^. This is, to the best of our knowledge, the first time a sub‐60‐mV dec^−1^ SS has been observed in MoS_2_/WSe_2_ heterostructures. Furthermore, we expect to achieve a higher ON current and a smaller SS over a larger current range in our future devices, by utilizing thinner high‐k dielectrics, further eliminating the resist contamination during the flake transfer to reduce the trap density at MoS_2_/HfO_2_ interface, or improving the contact at the MoS_2_ side.

**Figure 4:**
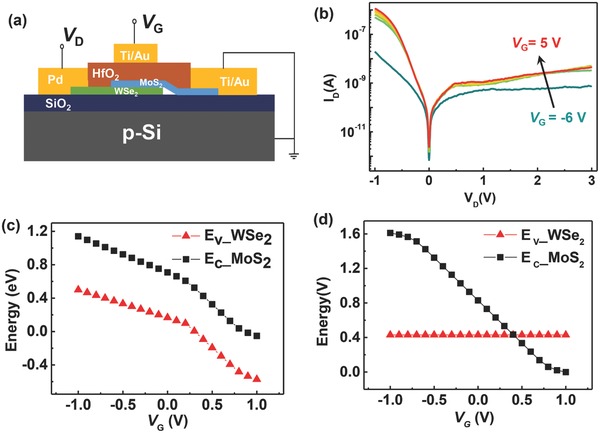
a) Schematic of a traditional MoS_2_/WSe_2_ heterostructure device. b) Output characteristics with *V*_G_ swept from −6 to 5 V, with an increment step of 3 V. c,d) Calculated tendency of MoS_2_ conduction band and WSe_2_ valence band as a function of *V*_G_ for the traditional and improved heterostructure devices, respectively.

In order to verify the superiority of our proposed device structure (type‐II), a control experiment was carried to compare the different device structures. The traditional device without a screening layer (type‐I) is schematically shown in **Figure** **4**a. To fabricate this type of device, we also confine the top‐gate electrode to cover the overlap region of MoS_2_/WSe_2_ heterostructure for the convenience of subsequent comparison and modeling. The output characteristics of the type‐I device are shown in Figure 4b. Apparently, its gate dependence under a negative *V*_D_ is better than that of forward *V*_D_ because the Au–MoS_2_ interface has a larger Schottky barrier (under positive *V*_D_, electrons are injected from Au to MoS_2_), which leads to a lower injection current. More importantly, the BTBT onset voltage *V*_ON_ is independent of *V*_G_, indicating a weak gate‐control capability of the relative band alignment for MoS_2_/WSe_2_, which also confirms the advantage of the type‐II device structure. Results from a simple model also support the experimental comparison results (details are discussed in the Supplementary Information). For two types of devices, the shifts of the MoS_2_ conduction band and the WSe_2_ valence band under different *V*_G_ values are shown in Figure 4c,d, respectively. In the type‐I device, MoS_2_ and WSe_2_ follow the same trend, and the relative band alignment is almost independent of *V*_G_, in agreement with the observed constant *V*_ON_; In the type‐II device, the WSe_2_ potential is pinned by the metallic screening layer, and thus the relative band alignment can be effectively modulated by *V*_G_, which agrees with the above experimental results.

**Figure 5:**
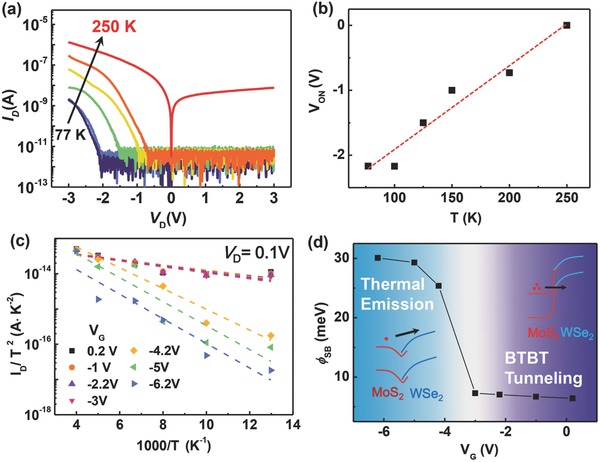
a) Temperature‐dependent *I*_D_–*V*_D_ characteristics measured at *V*_G_ = −9 V. b) Extracted *V*_ON_ versus *T. V*_ON_ is defined as the reverse *V*_D_ when *I*_D_ is higher than 100 pA. c) Arrhenius plot of temperature dependence for the *I*_D_ with varying *V*_G_; *V*_D_ = 0.1 V. d) Extracted effective Schottky‐barrier height φ_SB_ as a function of *V*_G_.

To obtain a more comprehensive understanding of the proposed heterostructure device, the temperature dependence of the output characteristics is also investigated. **Figure** **5**a plots the temperature‐dependent *I*_D_–*V*_D_ characteristics with *V*_G_ = −9 V, at *T* between 77 and 250 K. Figure 5b shows that *V*_ON_ is positively correlated with *T*. The extracted coefficient of 1.29 mV K^−1^ is also similar to the previous result.^24^ We then compare the datasets in Figure 5a with conventional thermal emission theory, which can be described by

(1) $$I_{D}=\;AT^{2}\exp\left(\frac{q\phi_{B}}{k_{B}T}\right)\left[1-\exp\left(\frac{qV_{D}}{k_{B}T}\right)\right]$$

where φ_B_ is the effective Schottky‐barrier height, *A* is the Richardson's constant, *k*_B_ is the Boltzmann's constant, and *q* is the electronic charge. An adequate fit to this equation can be obtained in Figure 5c, which shows an Arrhenius plot of *I*_D_/*T* ^2^ versus 1/*T* for different *V*_G_ values. The Schottky‐barrier height φ_B_ between MoS_2_ and WSe_2_ is then extracted and shown in Figure 5d. A clear transition of φ_B_ from 30 to 5 meV corresponds to the transition from the thermal‐emission‐dominated regime (*J*_Ther_) to the BTBT‐dominated regime (*J*_BTBT_), since the BTBT tunneling current contributes an additional current component that is not included in Equation (1). This also agrees with the underlying transport mechanism of the current fluctuation shown in Figure 3b.

**Figure 6:**
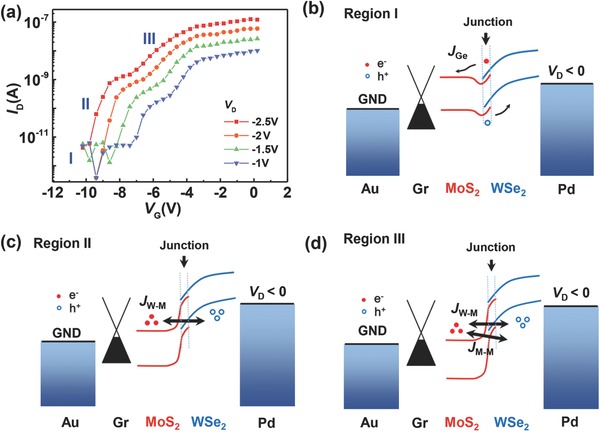
a) Transfer characteristics under negative *V*_D_ at 77 K, showing an obvious gate‐dependent current fluctuation. b–d) Corresponding schematic band diagrams for three different regions labeled in (a).

At relatively low temperature (from 77 to 200 K), we observe an unexpected “kink” signature in *I*_D_–*V*_G_ under negative *V*_D_, as shown in **Figure** **6**a. Both “kink” signatures and *V*_ON_ shift by varying *V*_D_. At higher *T* (>200 K), this phenomenon disappeared, as discussed in the Supplementary Information. Such an effect can be attributed to the combination of two BTBT processes: 1) BTBT from the WSe_2_ valence band to the MoS_2_ conduction band, and 2) BTBT occurring inside the MoS_2_ channel, since part of the MoS_2_ is screened by the palladium film.^44^ We define three different operation regions (I, II, and III) with corresponding band diagrams (Figure 6b–d) to further elucidate the underlying transport mechanism in each operation region. For large negative *V*_G_ in region I, both electron and hole diffusion currents are suppressed by energy barriers, showing an OFF state. In this region, the electrical field at the heterostructure region can rapidly pull out thermally excited electrons in the MoS_2_ conduction band and holes in the WSe_2_ valence band, to form a generation current (noted by *J*_Ge_ in Figure 6b). In region II, the MoS_2_‐conduction band can be pulled below the WSe_2_ valence band by *V*_G_ to form a BTBT window. In this case, the current is dominated by BTBT current from WSe_2_ to MoS_2_ (denoted *J*_W‐M_ in Figure 6c,d), and soon reaches saturation near the “kink” point as shown in Figure 6a. The BTBT current is positively proportional to applied *V*_D_ as the BTBT window can be enlarged by increasing the bias voltage, which can be quantitatively described by^45^

(2) $$J_{\mathrm{BTBT}}=\frac{2\pi \alpha q}{h}\;\;\int DOS_{M}\left(E\right)DOS_{W}\left(E\right)\left[f_{M}\left(E\right)-f_{W}\left(E-q\left(V_{D}-IR_{S}\right)\right)\right]dE$$

where α is the screening factor, *q* is the elementary charge, *h* is the Planck's constant, *V*_D_ is the bias voltage, *R*_s_ is the series resistance, and *DOS*_M_(*E*), *DOS*_W_(*E*), *f*_M_(*E*), and *f*_W_(*E*) represent the DOS and Fermi–Dirac distribution functions of MoS_2_ and WSe_2_, respectively. Equation (2) illustrates why the position of the “kink” feature shifts along with *V*_D_. If we continuously increase *V*_G_, the further relative band shift results in extra electrons in the BTBT current from the valence band of MoS_2_ underneath WSe_2_ (pinned by the screening layer) to the conduction band of MoS_2_ modulated by the top gate, as shown in Figure 6d and denoted as *J*_M‐M_. Therefore, a second enhancement of current is observed in region III. As the bandgap of MoS_2_ is larger than that of WSe_2_, the WSe_2_/MoS_2_ BTBT current appears prior to the MoS_2_/MoS_2_ BTBT current. Thus, we can observe the transition from WSe_2_/MoS_2_ BTBT current to MoS_2_/MoS_2_ BTBT current.

## 4 Conclusion

In summary, we have successfully demonstrated that a top‐gated MoS_2_/WSe_2_ vdWs heterostructure can be improved by inserting an extra self‐aligned metal film on top of the WSe_2_ portion as a screening layer. By tuning the bias voltage, this proposed device can be operated as a QED and UZD with obvious gate‐controlled current and on/off ratio. Under the BTBT regime, a 90% gate‐coupling efficiency, as well as a small SS (≈ 52 mV dec^− 1^ over two decades), is obtained owing to the effective and independent gate modulation on energy band and carrier density of the MoS_2_. By comparing with a traditional heterostructure, the superiority of the proposed device architecture is confirmed. At low temperature, a current fluctuation under negative *V*_D_ is also observed, and can be qualitatively explained by the alternation between WSe_2_/MoS_2_ BTBT current and MoS_2_/MoS_2_ BTBT current. Our proposed device architecture is versatile and can be conveniently applied in other 2DLM‐based heterostructures, and thus it is instrumental in designing future electronic and optoelectronic device applications.

## 5 Experimental Section

The WSe_2_ flake was first peeled onto the SiO_2_ (300 nm)/Si substrate by a mechanical exfoliation method. Electron‐beam lithography (EBL) was used to expose an area within the WSe_2_ flake (the area was slightly smaller than the flake itself), followed by 50‐nm Pd deposition using E‐beam evaporation. The self‐aligned dry etching was then performed using plasma etching (Ar gas, 40 sccm, 500 W) to obtain a sharp edge, which was the key to obtaining a working device. During the etching process, the Pd film was used as the protection layer and the rest of WSe_2_ was completely removed. Then, a dry transfer method^46^ was applied to transfer the Pd/WSe_2_ stack onto a MoS_2_ flake, which was pre‐deposited onto the SiO_2_ (300 nm)/Si substrate, to form a MoS_2_/WSe_2_ heterostructure. To improve the electrical contact at the MoS_2_ side, a graphene flake was attached to the MoS_2_ portion by the same dry transfer method. The source–drain electrodes were then defined by EBL followed by metal deposition (5 nm/50 nm Ti/Au). For the top‐gate dielectric, a 1‐nm‐thick layer of yttrium was thermally evaporated and then naturally oxidized in ambient air as a seeding layer for the following atomic layer deposition of high‐k HfO_2_ (50 nm) at 300 °C. Finally, a Ti/Au (5 nm/50 nm) layer was deposited as the top‐gate electrode.

## Conflict of Interest

The authors declare no conflict of interest.

## Supporting information

Supplementary
