# Supplementary material for: Independent Band Modulation in 2D van der Waals Heterostructures via a Novel Device Architecture
Source: Adv Sci (Weinh). 2018 Aug 2;5(9):1800237. doi: 10.1002/advs.201800237 (PMC6145257; doi:10.1002/advs.201800237)
Supplement: Supplementary file 1 — Supplementary [file ADVS-5-1800237-s001.pdf]

Copyright WILEY-VCH Verlag GmbH & Co. KGaA, 69469 Weinheim, Germany, 2018.

ADVANCED SCIENCE
Open Access

Supporting Information

for *Adv. Sci.*, DOI: 10.1002/advs.201800237

Independent Band Modulation in 2D van der Waals Heterostructures via a Novel Device Architecture

Zhongxun Guo, Yan Chen, Heng Zhang, Jianlu Wang, Weida Hu, Shijin Ding, David Wei Zhang, Peng Zhou, and Wenzhong Bao**

Supporting Information

Independent Band Modulation in Two-Dimensional van der Waals Heterostructure Based on a New TFET Device Architecture

Zhongxun Guo[1], Yan Chen[2], Heng Zhang[1], Jianlu Wang[2], Weida Hu[2], Shijin Ding[1], David Wei Zhang[1], Peng Zhou[1]*and Wenzhong Bao[1]*

[1]State Key Laboratory of ASIC and System, School of Microelectronics, Fudan University, Shanghai 200433, China

[2]State Key Laboratory of Infrared Physics, Shanghai Institute of Technical Physics, Chinese Academy of science, 500 Yutian Road, Shanghai 200083, China

S1: Raman spectroscopy

Figure S1 shows the Raman spectrum of exfoliated MoS_2, WSe_2 and graphene sheets. The optical images are also shown in the insets. A depolarized laser with 514-nm wavelength is focused at the red points (around 1um) marked in each inset. **Figure S1a** shows that the E_{2g} and A_{1g} peaks of the MoS_2 sheet locate at 383 cm^{-1} and 407 cm^{-1}, corresponding to 3-5 layers[1]. In **Figure S1b**, the E_{2g} peak of the WSe_2 sheet locates at 251cm^{-1} , corresponding to 3-5 layers[2]. For the graphene sheet, the G and 2D peaks locate at 1579 cm^{-1} and 2690 cm^{-1}, and can be identify as bilayer graphene [3].

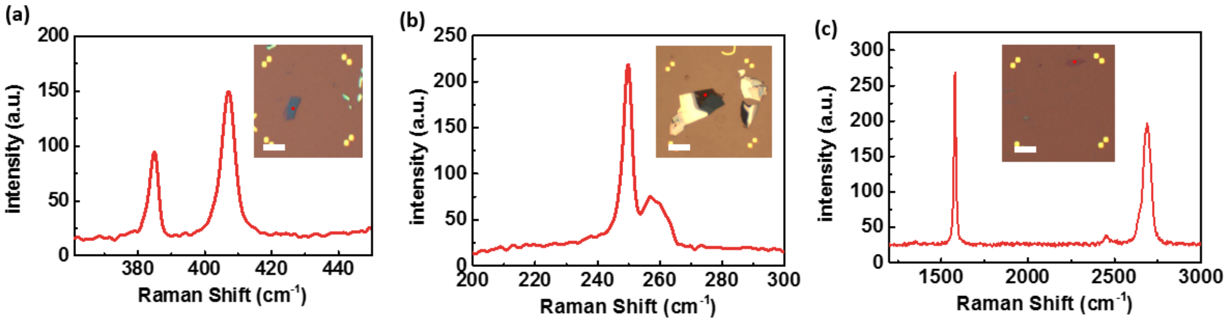

Figure S1 (a-c) Raman spectrum of MoS_2, WSe_2 and graphene, repectively. Optical image and laser focus point are shown in insets.

S2: Vertical band diagram sketch of the device

Figure S2a shows the schematic diagram of vertical energy band of our device. Pd inserted between WSe_2 and top gate dioxide performs as screening layer and also induces highly p-doping in the WSe_2 portion. **Figure S2b** shows the schematic diagram of vertical energy band of traditional MoS_2/WSe_2 heterostructure device.

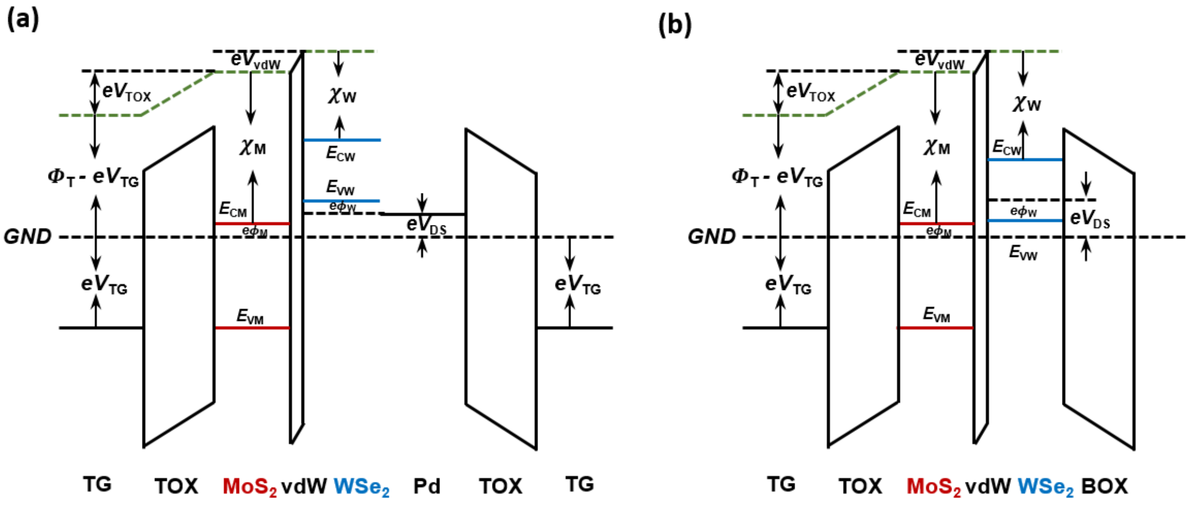

Figure S2 (a-b) Sketch of the vertical energy diagram for the Pd-WSe2/MoS_2 and traditional MoS_2/WSe_2 heterostructure devices, respectively.

S3: Theoretical model for calculation of calculating energy alignment

For our device, the corresponding band diagram is sketched in **Figure S2**. We use Guass's Law to correlate charge density and potential:[4]

$$C_{\mathrm{TOX}}V_{\mathrm{TOX}} - C_{\mathrm{vdW}}V_{\mathrm{vdW}} = -e\,(p_{\mathrm{M}} - n_{\mathrm{M}} + N_{\mathrm{DM}}) \qquad \text{S1}$$

$$C_{\mathrm{vdW}}V_{\mathrm{vdW}} = -e\,(p_{\mathrm{W}} - n_{\mathrm{w}}) \qquad \text{S2}$$

where C_{TOX} , V_{TOX} , C_{vdW} and V_{vdW} represent the capacitance and potential drop of the top oxide layer and vdW gap between MoS_2 and WSe_2, respectively; $p_{\mathrm{M}}, n_{\mathrm{M}}, p_{\mathrm{W}}, n_{\mathrm{w}}$ represent the hole and electron density of MoS_2 and WSe_2, respectively; N_{DM} is the donor density in MoS_2 due to the sulfur atom vacancy[5]. V_{TOX} and V_{vdW} can be obtained from vertical energy band diagram described by following equations:

$$\Phi_{\mathrm{T}} - eV_{\mathrm{TG}} + eV_{\mathrm{TOX}} = \chi_{\mathrm{M}} + e\phi_{\mathrm{M}} \quad \text{S3}$$

$$\chi_{\mathrm{M}} + e\phi_{\mathrm{M}} + eV_{\mathrm{vdW}} = \chi_{\mathrm{W}} + E_{\mathrm{GW}} - e\phi_{\mathrm{W}} + eV_{\mathrm{D}} \quad \text{S4}$$

where $e\phi_{\mathrm{M}} = E_{\mathrm{CM}} - E_{\mathrm{FM}}$, $e\phi_{\mathrm{M}} = E_{\mathrm{CW}} - E_{\mathrm{FW}}$, E_{FM} and E_{FW} represent the Fermi energy of MoS_2 and WSe_2, respectively; Φ_{T} is the work function of top metal gate ; χ_{M}, χ_{W} represent the electron affinity energy in MoS_2 and WSe_2 , respectively; and E_{GW} is the band gap of WSe_2。The potential drop within MoS_2 and WSe_2 can be negligible and both materials maintain thermodynamic equilibrium. Therefore, the Fermi energy in MoS_2 and WSe_2 are separated by bias voltage: $E_{\mathrm{FW}} - E_{\mathrm{FM}} = eV_{\mathrm{D}}$. The carrier density in two dimensional semiconductor can be given by[6]:

$$n_{\mathrm{M}} = \frac{g_{\mathrm{vM}} m_{\mathrm{c}} k_{\mathrm{B}} T}{\pi\hbar} \ln[\exp\left(-\frac{q\phi_{\mathrm{M}}}{k_{\mathrm{B}} T}\right) + 1] \quad \text{S5}$$

$$p_{\mathrm{M}} = \frac{g_{\mathrm{vW}} m_{\mathrm{v}} k_{\mathrm{B}} T}{\pi\hbar} \ln[\exp\left(-\frac{q\phi_{\mathrm{w}}}{k_{\mathrm{B}} T}\right) + 1] \quad \text{S6}$$

where g_{vM} and g_{vW} are valley degeneracies of MoS_2 conduction band and WSe_2 valance band, respectively.

By substituting equation S5-6 into S3-4, together with equations S1-2 we can numerically solve $V_{\mathrm{vdW}}, V_{\mathrm{TOX}}, \phi_{\mathrm{M}}, \phi_{\mathrm{W}}$.

S4: Output characteristics of CVD MoS_2 transistor with Pd contact.

Figure S4 shows output characteristics of a regular monolayer MoS_2 FET with Pd contact. Compared to the output characteristics of the device shown in Fig. 2a, the performance of this device is obviously different. It verifies the importance of the Pd-doped WSe_2 in our heterostructure device, which performs as a p-type component to suppress the reversed I_D and further facilitate the BTBT current.

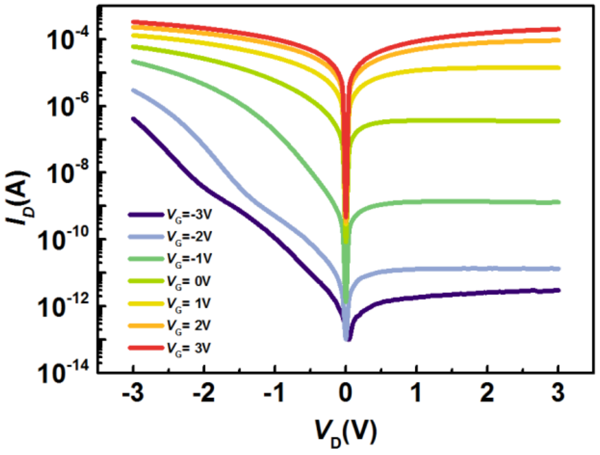

Figure S4 (a) Output characteristics of a monolayer MoS_2 FET with Pd contact. The V_G changes from -3V to 3V.

S5: Evolution of the "kink" feature at different temperatures.

Figure S5 shows transfer characteristics with negative V_D at different temperatures. The "kink" feature gradually fades with raised T and disappears as T >200K, which can be attributed to the broadening of DOS and distribution function at higher T.

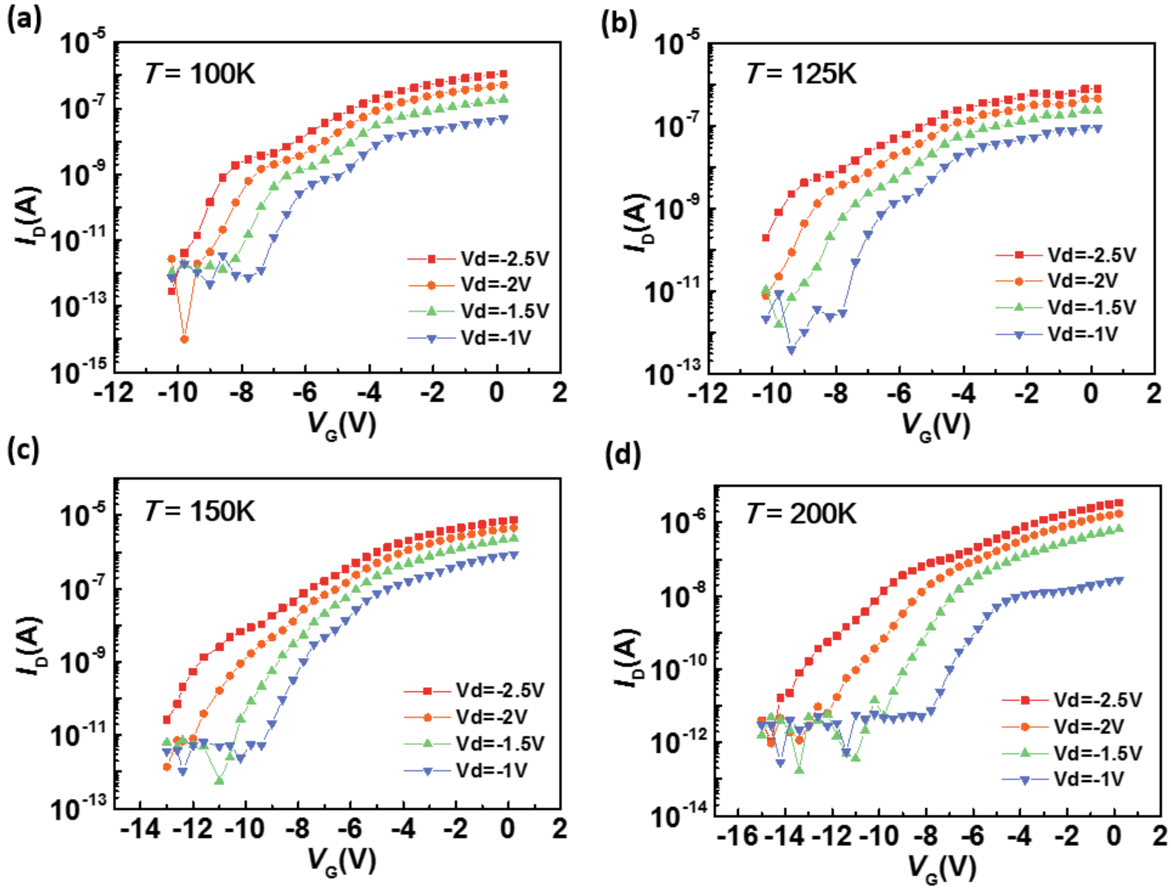

Figure S5 (a-d) Transfer characteristics with different negative values of V_D, measured at 100K, 125K, 150K and 200K, respectively.

References

1. Li, H.; Zhang, Q.; Yap, C. C. R.; Tay, B. K.; Edwin, T. H. T.; Olivier, A.; Baillargeat, D., From Bulk to Monolayer MoS2: Evolution of Raman Scattering. *Advanced Functional Materials* **2012,** *22* (7), 1385-1390.

2. Terrones, H.; Corro, E. D.; Feng, S.; Poumirol, J. M.; Rhodes, D.; Smirnov, D.; Pradhan, N. R.; Lin, Z.; Nguyen, M. A. T.; Elías, A. L., New first order Raman-active modes in few layered transition metal dichalcogenides. *Scientific Reports* **2014,** *4* (7489), 4215.

3. Malard, L. M.; Pimenta, M. A.; Dresselhaus, G.; Dresselhaus, M. S., Raman spectroscopy in graphene. *Physics Reports* **2009,** *473* (5–6), 51-87.
4. Li, M. O.; Esseni, D.; Nahas, J. J.; Jena, D.; Xing, H. G., Two-Dimensional Heterojunction Interlayer Tunneling Field Effect Transistors (Thin-TFETs). *IEEE Journal of the Electron Devices Society* **2015,** *3* (3), 200-207.
5. Hong, J.; Hu, Z.; Probert, M.; Li, K.; Lv, D.; Yang, X.; Gu, L.; Mao, N.; Feng, Q.; Xie, L., Exploring atomic defects in molybdenum disulphide monolayers. *Nature Communications* **2015,** *6*, 6293.
6. Li, M.; Esseni, D.; Snider, G.; Jena, D.; Xing, H. G., Single particle transport in two-dimensional heterojunction interlayer tunneling field effect transistor. *Journal of Applied Physics* **2014,** *115* (7), 074508-074508-11.
